# Association of perchlorate, nitrate, and thiocyanate with age-related macular degeneration in the United States

**DOI:** 10.1371/journal.pone.0334919

**Published:** 2025-10-29

**Authors:** Shiyu Jia, Qin Liu, Peng Liu, Wenli Zhao, Shanshan Li

**Affiliations:** 1 Department of Ophthalmology, Workers’ Hospital of Jinchuan Group Co., Ltd., Jinchang, Gansu, China; 2 Gansu Aier Eye Hospital, Lanzhou, Gansu, China; 3 Gansu Provincial Hospital, Lanzhou, Gansu China; 4 Neonatal Intensive Care Unit, Workers’ Hospital of Jinchuan Group Co., Ltd., Jinchang, Gansu, China; West Bengal University of Animal and Fishery Sciences, INDIA

## Abstract

Perchlorate, nitrate, and thiocyanate are endocrine-disrupting chemicals, but their associations with AMD is unclear. This study aims to investigate this relationship. We included 4727 participants aged 40 years and older from the National Health and Nutrition Examination Survey (NHANES) 2005–2008. Logistic regression analysis, restricted cubic spline (RCS), and weighted quantile sum (WQS) were applied to investigate the single, non-linear, and combined effects on AMD risk. Nitrate exposure was positively associated with any AMD risk (OR _Any AMD_, 1.19; 95% CI, 1.05–1.35; P = 0.010) and early AMD risk (OR _Early AMD_, 1.19; 95% CI, 1.05–1.36; P = 0.010); compared to the first quartile, the highest quartile of nitrate (OR, 1.94; 95% CI, 1.18–3.19; P = 0.012) and thiocyanate (OR, 1.70; 95% CI, 1.19–2.42; P = 0.006) levels were positively associated with AMD risk. The results of RCS showed a nonlinear relationship between nitrate exposure (P for nonlinearity = 0.020), thiocyanate (P for nonlinearity = 0.041), and AMD risk. WQS analysis indicated a positive relationship between mixed exposure and AMD risk (OR, 1.24; 95% CI, 1.01 to 1.51; P = 0.037). This study indicated that high urinary nitrate and thiocyanate levels were associated with an increased AMD risk among US adults. However, the cross-sectional design precludes causal inference.

## 1. Introduction

Age-related macular degeneration (AMD) is a main cause that leads to the visual impairment that mainly affects the elderly in developed countries, with an estimated 196 million cases in 2020 projected to rise to 288 million by 2040 [1,2]. AMD is promoted by multiple factors [3]. Although, researchers have revealed many risk factors of AMD including advanced age [4] and eating habits [5], the etiology of AMD has not been fully illustrated. Recently, many environmental factors were found increased AMD risk, such as cadmium, nitrogen dioxide, and per- and polyfluoroalkyl substances exposure [6–8].

Perchlorate, nitrate, and thiocyanate are three common inorganic anions, which can interfere with the function of the thyroid through competitively inhibiting the sodium iodide symporter (NIS)-mediated intake of iodide [9]. Perchlorate, as an environmental pollutant, is commonly used in industrial production such as fertilizer, fireworks, and rocket fuel [10]. Nitrate widely exists in leafy vegetables, cured meats, and contaminated water [11]. Thiocyanate is mainly present in the electronic industry and the metabolites of cigarette smoke [9,12]. Epidemiological studies have provided plenty of evidence of the association between AMD with thyroid dysfunction, including hypothyroidism and hyperthyroidism [13–15]. Many studies have reported the toxicity of perchlorate, nitrate, and thiocyanate on the thyroid [16,17]. Except for thyroid function, the effects of these chemicals were controversial on many other health outcomes, such as dyslipidemia [18], obesity [19], and cardiovascular diseases [20]. Biologically, nitrate may influence nitric oxide (NO)-related vascular processes [21], thiocyanate is closely tied to tobacco exposure and oxidative pathways, and perchlorate competitively inhibits the NIS, potentially perturbing the thyroid axis [9].

However, the relationship between nitrate exposure and AMD risk remained controversial. A previous study found that higher nitrate-nitrogen concentrations in drinking water was correlated with a higher incidence of AMD [22]. However, another study has reported that high dietary nitrate intake is associated with a lower incidence of early AMD [23]. A recent study has also revealed the negative association between dietary nitrate intake with late AMD risk, but this relationship was not significant after adjusting for the Mediterranean diet [24]. It is difficult to investigate a single nutrient consumption on the risk of a disease, because an individual usually takes in hundreds of nutrients per day and this may lead to bias. Urinary concentrations of these three chemicals were considered reliable biomarkers for assessing the status of human exposure [25,26].

Given the lack of evidence of the correlation between perchlorate or thiocyanate with AMD, and controversial results of the association between nitrate with AMD, we intend to detect whether urinary thiocyanate, perchlorate and nitrate increased AMD risk using US samples.

## 2. Methods

### 2.1 Data collection

The study obtained data from the US National Health and Nutrition Examination Survey (NHANES), a cross-section series of interviews and examinations of the civilian, noninstitutionalized US individuals. All participants have achieved informed consent. The National Center for Health Statistics board approved the protocols, so this study did not need to provide additional ethics approval.

### 2.2 Measurements of exposure

NHANES staff collected urinary samples. The urinary samples were processed, stored at −20°C, and then sent to the CDC for further analysis. Chromatographic separation is conducted using an IonPac AS16 column with sodium hydroxide as the eluant. Detailed procedures can be found on the official website (https://wwwn.cdc.gov/Nchs/Nhanes/2005-2006/PERNT_D.htm).

### 2.3 Retinal photography and AMD grading

In the NHANES 2005–2008 cycles, fundus images were photographed through Canon CR6–45NM system. The presence of AMD was assessed by two specialists [27,28]. Early AMD was defined by the presence of pigmentary abnormalities and/or soft drusen with a grid area larger than a 500 μm circle, whereas the existence of geographic atrophy (GA), the subretinal fibrous scar of choroidal neovascularization was defined as late AMD.

### 2.4 Covariates

The following covariates were considered according to previous AMD literature and NHANES variables: age, income-poverty ratio (PIR), gender, body mass index (BMI, kg/m^2^), race, urinary creatinine (mg/dL), serum HDL levels (mg/dL), smoking, drinking, and self-reported hypertension, diabetes, and cardiovascular disease.

### 2.5 Statistical analysis

We conducted analyses following the CDC guidelines for NHANES data [29]. Characteristics were displayed as means ± standard deviation (SD) or percentages. Because of the skewed distribution of urinary perchlorate, nitrate, and thiocyanate, log-2 transformed concentrations replaced the original values to avoid the effects of extreme values. Meanwhile, the original concentrations were analyzed in quartiles. We calculated Pearson correlation coefficients among urinary perchlorate, nitrate, and thiocyanate to evaluate potential correlations between exposures. Four logistic regression models were used to calculate odds ratios (OR) and 95% confidence intervals (CI). Model 1 was adjusted for age and urinary creatinine. Model 2 was adjusted for age, sex, race, PIR, BMI and urinary creatinine. Model 3 was further adjusted for HDL, smoking, alcohol drinking, hypertension, diabetes and cardiovascular diseases based on model 2. Restricted cubic spline (RCS) was used to detect the non-linear relationship between Log2-transformed perchlorate, nitrate, and thiocyanate and the risk of AMD using three defined knots (5th, 50th, and 95th percentiles). Subgroup analyses were performed according to the covariates. The weighted quantile sum (WQS) regression analysis was performed for the three chemicals, using gWQS package of R software [30]. Besides, three sensitive analyses were conducted to validate the robustness of our results. First, we used multiple imputation to deal with missing covariates values (sensitivity ⅰ) [31]. Second, after excluding those participants with extreme values (more than 99% or less than 1%), original urinary perchlorate, nitrate, and thiocyanate levels were used for analysis (sensitivity ⅱ). Third, Mediterranean diet index and serum cadmium were added into the model 3 for analysis (sensitivity ⅲ). Data accounted for NHANES weights was analyzed using R 4.3.2. Statistically significance: P < 0.05.

## 3. Results

### 3.1 Characteristics

**Fig 1:**
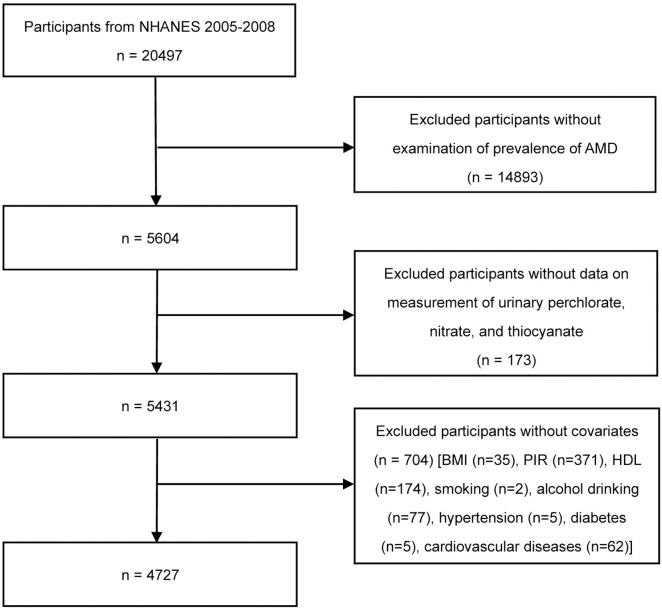
Selection of Study population.

A total of 20,497 participants from NHANES 2005–2008 were enrolled. We removed those participants who had missing data on the diagnosis of AMD (n = 14893) and urinary levels of perchlorate, nitrate, and thiocyanate (n = 173). Then, participants who had unavailable covariates were excluded (n = 704) [BMI (n = 35), PIR (n = 371), HDL (n = 174), smoking (n = 2), alcohol drinking (n = 77), hypertension (n = 5), diabetes (n = 5), cardiovascular diseases (n = 62)] (**Fig 1**). In addition, participants included in the analysis were older and more likely to be non-Hispanic White than those excluded (P < 0.05, S1 Table).

**Fig 2:**
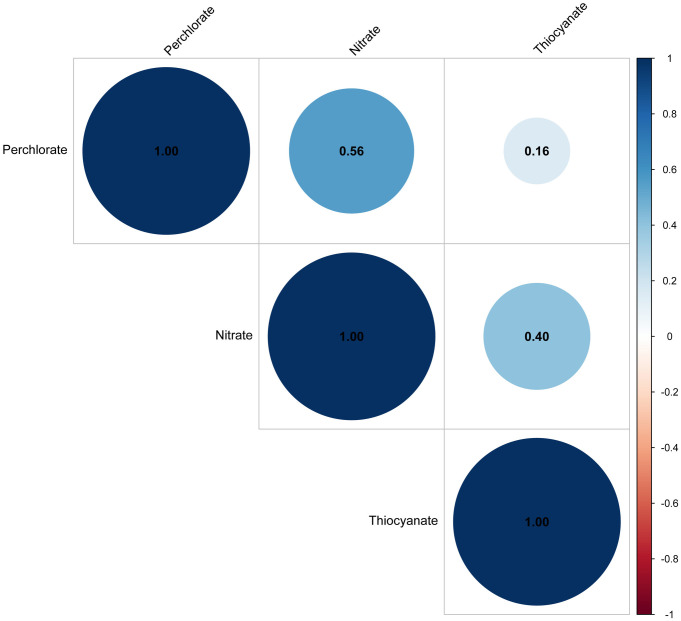
Pearson correlation coefficients among study variables.

Finally, 4727 participants aged ≥ 40 years were available for analyses, of which 362 were AMD and 4365 were non-AMD individuals. The prevalence of AMD was 7.7%. Among 362 AMD patients, 319 were early AMD and 43 were late AMD. The mean age was 55.9 ± 11.4 years. 52.0% of participants were females. Compared with non-AMD individuals, AMD patients were prone to be older, non-Hispanic White, having higher levels of HDL-C, and drinkers (P < 0.05). In addition, AMD patients had a higher prevalence of hypertension and cardiovascular diseases (P < 0.01) (**Table 1**). Pearson correlation analysis showed moderate correlations between perchlorate and nitrate (r = 0.56) and between nitrate and thiocyanate (r = 0.40), while the correlation between perchlorate and thiocyanate was weaker (r = 0.16) (Fig 2).

**Table 1:** Demographics and health status of participants with and without AMD.

	All participants (n = 4727)	Non-AMD (n = 4365)	All AMD (n = 362)	P-value
Age, mean (SD), y	55.93 (11.43)	55.18 (10.95)	67.13 (12.63)	<0.001
Sex, No. (%)				0.950
Male	2397 (48.0)	2207 (48.0)	190 (48.2)	
Female	2330 (52.0)	2158 (52.0)	172 (51.8)	
Race, No. (%)				<0.001
Non-Hispanic White	2604 (78.2)	2348 (77.7)	256 (86.1)	
Non-Hispanic Black	925 (8.9)	891 (9.2)	34 (4.2)	
Others	1198 (12.9)	1126 (13.1)	72 (9.7)	
BMI, kg/m^2^	29.08 (6.45)	29.11 (6.48)	28.55 (6.05)	0.208
PIR, No. (%)				0.372
>1	714 (8.7)	661 (8.6)	55 (9.7)	
≥1	4011 (91.3)	3704 (91.4)	307 (90.3)	
Education level, No. (%)				0.180
High school graduate and below	1319 (17.1)	1219 (16.9)	100 (20.9)	
High school graduate	3408 (82.9)	3146 (83.1)	262 (79.1)	
HDL-C, mean (SD), mg/dL	53.78 (16.22)	53.59 (16.11)	56.59 (17.60)	0.002
Smoking, No. (%)	2493 (51.5)	2298 (51.2)	195 (54.8)	0.333
Alcohol drinking, No. (%)	3259 (73.0)	3022 (73.4)	237 (66.5)	0.027
Hypertension, No. (%)	2119 (40.7)	1919 (40.0)	200 (51.1)	0.009
Diabetes, No. (%)	659 (10.0)	612 (9.9)	47 (11.6)	0.352
Cardiovascular diseases, No. (%)	670 (11.0)	568 (10.0)	102 (25.8)	<0.001
Perchlorate (median [IQR]) *	3.69 (2.33, 6.00)	3.68 (2.31, 5.99)	4.02 (2.53,.6.07)	0.128
Nitrate (median [IQR]) *	44550.08 (31671.48, 64278.38)	44482.76 (31585.37, 63998.21)	46499.97 (34205.68, 68400.79)	0.075
Thiocyanate (median [IQR]) *	1301.59 (639.05, 2889.76)	1302.52 (647.17, 2900.20)	1259.84 (538.60, 2684.19)	0.372

Abbreviations: BMI, body mass index; PIR, income-poverty ratio; HDL-C, high-density lipoprotein cholesterol. ⁎ unit: μg/g creatinine.

### 3.2 Association of perchlorate, nitrate, and thiocyanate with AMD risk

Log2-transformed urinary nitrate level was positively associated with AMD risk (OR, 1.19; 95% CI, 1.05–1.35; P = 0.010). After assigning original concentrations into quartiles, compared to the first quartile, the highest quartile of nitrate (OR, 1.94; 95% CI, 1.18–3.19; P = 0.012) and thiocyanate (OR, 1.70; 95% CI, 1.19–2.42; P = 0.006) levels were positively associated with AMD risk (**Table 2**), with significant trends across quartiles for both exposures (P for trend = 0.020 and 0.004, respectively).

**Table 2:** Logistic regression analysis of the association of perchlorate, nitrate, and thiocyanate exposure and AMD risk.

	Model 1		Model 2		Model 3	
	OR (95%CI)	P-value	OR (95%CI)	P-value	OR (95%CI)	P-value
Perchlorate, ng/mL						
Log2-transformed	1.00 (0.88, 1.14)	0.997	0.99 (0.86, 1.13)	0.857	0.98 (0.85, 1.13)	0.785
Quartile 1 (<1.87)	Reference					
Quartile 2 (1.87–3.48)	0.89 (0.62, 1.27)	0.511	0.88 (0.61, 1.26)	0.458	0.86 (0.59, 1.26)	0.419
Quartile 3 (3.48–6.11)	1.39 (0.99, 1.95)	0.057	1.35 (0.96, 1.89)	0.083	1.32 (0.93, 1.89)	0.110
Quartile 4 (≥6.11)	0.87 (0.54, 1.42)	0.576	0.83 (0.50, 1.38)	0.464	0.82 (0.48, 1.38)	0.429
P for trend	0.870		0.911		0.905	
Nitrate, ng/mL						
Log2-transformed	1.20 (1.06, 1.35)	0.005	1.19 (1.05, 1.35)	0.009	1.19 (1.05, 1.35)	0.010
Quartile 1 (<24700)	Reference					
Quartile 2 (24700–43200)	1.31 (0.85, 2.01)	0.205	1.31 (0.85, 2.01)	0.211	1.34 (0.86, 2.07)	0.171
Quartile 3 (43200–68900)	1.13 (0.71, 1.80)	0.599	1.10 (0.69, 1.75)	0.677	1.11 (0.68, 1.82)	0.645
Quartile 4 (≥68900)	1.94 (1.19, 3.15)	0.009	1.90 (1.15, 3.14)	0.015	1.94 (1.18, 3.19)	0.012
P for trend	0.013		0.023		0.020	
Thiocyanate, ng/mL						
Log2-transformed	1.07 (0.99, 1.16)	0.100	1.06 (0.98, 1.15)	0.149	1.07 (0.98, 1.16)	0.127
Quartile 1 (<566)	Reference					
Quartile 2 (566–1160)	1.17 (0.81, 1.68)	0.379	1.16 (0.80, 1.68)	0.417	1.16 (0.79, 1.71)	0.416
Quartile 3 (1160–2600)	1.30 (0.93, 1.82)	0.122	1.28 (0.90, 1.83)	0.159	1.28 (0.89, 1.84)	0.163
Quartile 4 (≥2600)	1.67 (1.23, 2.28)	0.002	1.64 (1.18, 2.27)	0.005	1.70 (1.19, 2.42)	0.006
P for trend	0.002		0.004		0.004	

Model 1 was adjusted for age and urinary creatinine

Model 2 was adjusted for age, sex, race, PIR, BMI and urinary creatinine.

Model 3 was adjusted for age, sex, race, PIR, BMI, HDL, smoking, alcohol drinking, hypertension, diabetes, cardiovascular diseases and urinary creatinine.

After stratifying by AMD subtypes, log2-transformed urinary nitrate level was positively associated with early AMD risk (OR, 1.19; 95% CI, 1.05–1.36; P = 0.010). After assigning original concentrations into quartiles, compared to the first quartile, the highest quartile of nitrate (OR, 1.93; 95% CI, 1.16–3.20; P = 0.014) and thiocyanate (OR, 1.66; 95% CI, 1.15–2.38; P = 0.010) levels were positively associated with early AMD risk, and the third quartile of thiocyanate level was positively associated with late AMD risk (OR, 2.53; 95% CI, 1.06–2.61; P = 0.039) (**Table 3**).

**Table 3:** The association of perchlorate, nitrate, and thiocyanate exposure and early and late AMD risk.

	Early AMD		Late AMD	
	OR (95%CI)	P-value	OR (95%CI)	P-value
Log2-transformed perchlorate, ng/mL	0.98 (0.84, 1.15)	0.803	0.97 (0.69, 1.36)	0.837
Quartile 1	Reference			
Quartile 2	0.84 (0.56, 1.27)	0.387	1.14 (0.28, 4.57)	0.849
Quartile 3	1.25 (0.84, 1.88)	0.250	1.97 (0.43, 9.03)	0.356
Quartile 4	0.79 (0.45, 1.41)	0.408	1.12 (0.25, 4.94)	0.878
Log2-transformed nitrate, ng/mL	1.19 (1.05, 1.36)	0.010	1.19 (0.84, 1.67)	0.303
Quartile 1	Reference			
Quartile 2	1.32 (0.84, 2.08)	0.213	1.51 (0.50, 4.62)	0.441
Quartile 3	1.05 (0.63, 1.74)	0.849	1.59 (0.64, 3.92)	0.294
Quartile 4	1.93 (1.16, 3.20)	0.014	1.99 (0.54, 7.37)	0.279
Log2-transformed thiocyanate, ng/mL	1.06 (0.97, 1.16)	0.206	1.16 (0.83, 1.62)	0.363
Quartile 1	Reference			
Quartile 2	1.18 (0.77, 1.81)	0.415	1.05 (0.44, 2.55)	0.893
Quartile 3	1.20 (0.79, 1.81)	0.364	2.53 (1.06, 2.61)	0.039
Quartile 4	1.66 (1.15, 2.38)	0.010	1.99 (0.38, 10.37)	0.389

Model was adjusted for age, sex, race, PIR, BMI, HDL, smoking, alcohol drinking, hypertension, diabetes, cardiovascular diseases and urinary creatinine.

**Fig 3:**
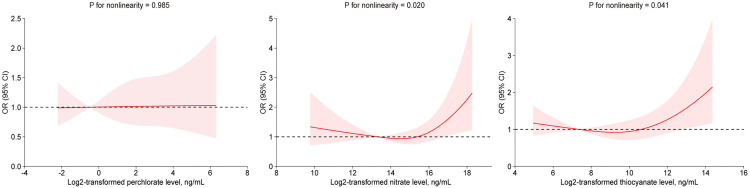
Dose-response relationship between log2-transformed urinary levels of perchlorate, nitrate, and thiocyanate with AMD. Note: The model was adjusted for age, sex, race, PIR, BMI, HDL, smoking, alcohol drinking, hypertension, diabetes, cardiovascular diseases and urinary creatinine.

Besides, after adjusting for all covariates, the results of RCS showed a U-shaped nonlinear relationship between log2-transformed nitrate (P for nonlinearity = 0.020), thiocyanate (P for nonlinearity = 0.041), and AMD risk (**Fig 3**).

### 3.3 WQS analysis

A positive connection between mixed perchlorate, nitrate, and thiocyanate exposure and AMD risk was detected by WQS analysis (OR, 1.24; 95% CI, 1.01 to 1.51; P = 0.037), of which nitrate accounted for 56.8% weights of the mixed exposure.

### 3.4 Subgroup analyses

The results of subgroup analyses were shown in **Table 4**. Log2-transformed nitrate level was positively associated with AMD risk among age ≥ 65 group (OR, 1.18; 95% CI, 1.02–1.36; P = 0.027), non-Hispanic White group (OR, 1.21; 95% CI, 1.07–1.37; P = 0.005), non-hypertension group (OR, 1.43; 95% CI, 1.11–1.84; P = 0.008), and non-diabetes subgroup (OR, 1.17; 95% CI, 1.00–1.35; P = 0.043). Significant interaction between nitrate and hypertension on AMD risk was detected (P for interaction = 0.041); log2-transformed thiocyanate level was positively associated with AMD risk among the diabetes subgroup (OR, 1.48; 95% CI, 1.16–1.89; P = 0.003). Significant interaction between thiocyanate and diabetes on AMD risk was detected (P for interaction = 0.025).

**Table 4:** Subgroup analysis.

	Perchlorate		P for interaction	Nitrate		P for interaction	Thiocyanate		P for interaction
	OR (95%CI)	P-value		OR (95%CI)	P-value		OR (95%CI)	P-value	
Age			0.796			0.212			0.126
<65	0.95 (0.78, 1.16)	0.604		1.26 (0.99, 1.60)	0.055		1.06 (0.91, 1.23)	0.435	
≥65	1.01 (0.84, 1.23)	0.879		1.18 (1.02, 1.36)	0.027		1.05 (0.93, 1.18)	0.412	
Sex			0.516			0.508			0.931
Male	1.03 (0.86, 1.22)	0.749		1.23 (0.97, 1.54)	0.081		1.05 (0.92, 1.20)	0.441	
Female	0.95 (0.79, 1.14)	0.565		1.17 (0.92, 1.49)	0.180		1.07 (0.92, 1.25)	0.389	
Race			0.355			0.588			0.802
White	0.97 (0.81, 1.16)	0.711		1.21 (1.07, 1.37)	0.005		1.08 (0.97, 1.19)	0.142	
Black	0.92 (0.63, 1.34)	0.646		0.87 (0.54, 1.40)	0.539		0.99 (0.74, 1.33)	0.976	
Others	1.08 (0.80, 1.45)	0.590		1.18 (0.87, 1.61)	0.261		1.01 (0.80, 1.28)	0.911	
Smoking			0.346			0.819			0.367
Yes	0.96 (0.81, 1.14)	0.644		1.18 (0.99, 1.42)	0.068		1.00 (0.91, 1.10)	0.979	
No	1.03 (0.81, 1.31)	0.788		1.19 (0.99, 1.42)	0.061		1.12 (0.99, 1.29)	0.077	
Alcohol drinking			0.491			0.955			0.551
Yes	0.95 (0.81, 1.10)	0.443		1.18 (0.97, 1.44)	0.088		1.01 (0.91, 1.13)	0.812	
No	1.08 (0.86, 1.35)	0.487		1.24 (0.92, 1.68)	0.151		1.16 (0.97, 1.38)	0.103	
Hypertension			0.110			0.041			0.445
Yes	0.93 (0.78, 1.11)	0.387		1.04 (0.88, 1.22)	0.636		1.05 (0.89, 1.24)	0.536	
No	1.03 (0.86, 1.22)	0.752		1.43 (1.11, 1.84)	0.008		1.07 (0.98, 1.17)	0.102	
Diabetes			0.957			0.682			0.025
Yes	1.20 (0.89, 1.62)	0.224		1.31 (0.91, 1.87)	0.135		1.48 (1.16, 1.89)	0.003	
No	0.96 (0.83, 1.13)	0.638		1.17 (1.00, 1.35)	0.043		1.03 (0.94, 1.13)	0.503	
Cardiovascular diseases			0.463			0.689			0.112
Yes	1.11 (0.87, 1.41)	0.386		1.22 (0.91, 1.63)	0.169		1.22 (1.03, 1.45)	0.027	
No	0.95 (0.82, 1.11)	0.484		1.16 (0.98, 1.39)	0.083		1.03 (0.92, 1.15)	0.621	

Model was adjusted for age, sex, race, PIR, BMI, HDL, smoking, alcohol drinking, hypertension, diabetes, cardiovascular diseases and urinary creatinine.

### 3.5 Sensitivity analyses

The results of sensitivity analyses also presented nitrate and thiocyanate could increase AMD risk (**Table 5**).

**Table 5:** Sensitivity analysis.

	Sensitivity i (n = 5431)		Sensitivity ii (n = 4444)		Sensitivity iii (n = 4273)	
	OR (95%CI)	P-value	OR (95%CI)	P-value	OR (95%CI)	P-value
Perchlorate						
Continuous	0.98 (0.87, 1.11)	0.779	0.97 (0.81, 1.15)	0.685	0.99 (0.85, 1.16)	0.931
Quartile 1	Reference					
Quartile 2	0.84 (0.56, 1.26)	0.370	0.82 (0.54, 1.24)	0.322	0.90 (0.59, 1.37)	0.605
Quartile 3	1.31 (0.93, 1.86)	0.117	1.35 (0.91, 2.02)	0.129	1.34 (0.89, 2.01)	0.142
Quartile 4	0.82 (0.53, 1.26)	0.339	0.77 (0.44, 1.35)	0.343	0.84 (0.47, 1.50)	0.524
Nitrate						
Continuous	1.17 (1.04, 1.32)	0.012	1.26 (1.05, 1.51)	0.016	1.22 (1.07, 1.39)	0.006
Quartile 1	Reference					
Quartile 2	1.24 (0.84, 1.85)	0.261	1.48 (0.90, 2.41)	0.111	1.31 (0.82, 2.11)	0.238
Quartile 3	1.14 (0.75, 1.74)	0.504	1.27 (0.76, 2.12)	0.346	1.11 (0.65, 1.92)	0.673
Quartile 4	1.70 (1.04, 2.80)	0.036	1.88 (1.14, 3.10)	0.016	1.94 (1.11, 3.40)	0.023
Thiocyanate						
Continuous	1.07 (0.99, 1.16)	0.082	1.11 (1.01, 1.22)	0.027	1.08 (0.98, 1.18)	0.127
Quartile 1	Reference					
Quartile 2	1.07 (0.76, 1.51)	0.661	1.32 (0.86, 2.03)	0.187	1.05 (0.65, 1.70)	0.813
Quartile 3	1.29 (0.92, 1.80)	0.132	1.28 (0.85, 1.95)	0.219	1.36 (0.90, 2.07)	0.130
Quartile 4	1.75 (1.25, 2.45)	0.003	1.85 (1.26, 2.71)	0.004	1.74 (1.23, 2.48)	0.005

Model was adjusted for age, sex, race, PIR, BMI, HDL, smoking, alcohol drinking, hypertension, diabetes, cardiovascular diseases, urinary creatinine (not in Sensitivity ⅱ), Mediterranean diet index (only in Sensitivity ⅲ) and serum cadmium (only in Sensitivity ⅲ).

Sensitivity ⅰ: Multiple imputation for covariates.

sensitivity ⅱ: After excluding those participants with extreme values (more than 99% or less than 1%), original urinary perchlorate, nitrate, and thiocyanate levels were used for analysis.

Sensitivity ⅲ: Mediterranean diet index and serum cadmium were added into the model for analysis.

## 4. Discussion

As far as we know, this is the first study to investigate the relationship between urinary concentrations of perchlorate, nitrate, thiocyanate, and AMD risk. The results indicated that high nitrate and thiocyanate concentrations were significantly correlated with a higher AMD risk especially early AMD risk, while perchlorate was not correlated with AMD risk. In addition, quartile analyses revealed significant positive trends across nitrate and thiocyanate levels, suggesting potential dose-response relationship. Although the OR for the highest quartiles indicate moderate association, such effect sizes may still be meaningful at the population level given the high prevalence of AMD and widespread exposure to these chemicals. Moreover, the RCS demonstrated a U-shaped relationship between nitrate, thiocyanate, and AMD risk. A positive connection between mixed perchlorate, nitrate, and thiocyanate exposure and AMD risk was revealed by WQS analysis. It should be noted that the WQS weights should be interpreted with caution. Although WQS regression is useful for evaluating mixture effects, it cannot fully disentangle true biological contributions from statistical dependence among correlated exposures. In our analysis, nitrate contributed more than half of the mixture effect, but this may partly reflect its correlations with thiocyanate and perchlorate rather than its independent effect. Therefore, the WQS weights should be viewed as relative indicators of contribution within the mixture rather than definitive measures of individual exposure effects.

A previous study has reported the negative association of dietary nitrate intake and AMD incidence [23]. Dietary vegetables consumption was the most important source of nitrate [32], and high dietary nitrate intake was usually associated with a healthier diet that could decrease the risk of AMD [33,34]. Interestingly, another study found that the negative association between dietary nitrate intake with AMD risk was not significant after further adjusted for Mediterranean dietary patterns [24]. It is hard to assess the effect of single nutrient intake on AMD risk because an individual usually takes in hundreds of nutrients per day. Our findings were consistent with a study assessing the relationship of nitrate-nitrogen levels in drinking water with AMD incidence [22]. In our study, only the extreme quartile of nitrate and thiocyanate concentrations increased AMD risk, and the RCS revealed the nonlinear relationship. These results demonstrated the threshold effects.

Few studies have explored the mechanism between nitrate and thiocyanate exposure on retinal function or AMD risk. Choroidal neovascularization (CNV) is the key characteristic of nAMD [35]. In vivo study, supplemental nitrate in drinking water significantly increased serum levels of nitrate and the volume of CNV in mice, indicating the adverse effects of nitrate intake [21]. In the human gut, nitrates in food or water are first metabolized to nitrites by reductase, and then transformed to NO [36]. The role of NO in the pathogenesis of AMD is not very clear [37]. In human bodies, another route of the generation of NO is the transformation of L-arginine to L-citrulline and NO [38]. Normally, endothelial NOS-derived NO has the ability of vascular relaxing [39]. However, high levels of NO can generate amounts of reactive nitrogen species such as peroxynitrite, a reactive tissue impairing substance, which can cause the accumulation of abnormal proteins and then lead to the damage of retinal pigment epithelial (RPE) cell and degeneration and apoptosis of photoreceptors [40,41]. The main routes of thiocyanate exposure are intake of cruciferous vegetables and cigarette smoking [42]. H2O2 can convert thiocyanate to hypothiocyanous acid by myeloperoxidase (MPO) and lactoperoxidase [43]. It is reported that high levels of thiocyanate could easily cross the basolateral membrane of RPE and impact the electrophysiological function of the retina [44]. Thiocyanate and MPO could be further delivered to the lysosomes of RPE cells and accumulation of MPO in lysosomes can trigger stress and lead to cell death [45,46], while lysosomes dysfunction plays a crucial role in the pathogenesis of early AMD [47]. In addition, patients with hypothyroidism are associated with a higher risk of AMD, the exact mechanism is unclear [13,15]. This study showed that in three NIS inhibitors, perchlorate was not correlated with AMD risk. Long-term exposure to nitrate and thiocyanate, but not perchlorate, has been associated with hypothyroidism in adults, this may be attributed to the low exposure levels of perchlorate worldwide [48,49]. Further experimental studies are needed to investigate the underlying mechanisms between nitrate and thiocyanate exposure with AMD risk

The null associations observed for perchlorate merit consideration. First, urinary perchlorate levels in NHANES are typically low than nitrate or thiocyanate, which may limit statistical power to detect modest effects. Second, perchlorate’s primary mode of action, competitive inhibition of the sodium and iodide symporter with downstream thyroid perturbation, may be less directly linked to AMD pathways than nitrate and thiocyanate-related mechanisms (NO-related CNV and MPO-mediated oxidative processes, respectively) [21].

Nevertheless, several limitations exist in this study that should be taken into rigorous consideration when explaining these results. First, the causality cannot be determined. Second, the reliance on single-spot urine samples may not fully capture temporal variation and could be influenced by recent diet or short-term exposures, potentially leading to exposure misclassification and attenuation of associations. However, a previous study has provided evidence of the high temporal reliability of urinary concentrations of perchlorate, nitrate, and thiocyanate, indicating they were reliable biomarkers of long-term exposure [50]. Third, residual confounding cannot be excluded. Although our models adjusted for multiple covariates, some other factors such as dietary patterns, supplement use, and other environmental co-exposures, may influence both urinary biomarker levels and AMD risk. In addition, clinical factors such as anti-VEGF treatment and other potential determinants not captured in our data could lead to the bias. Fourth, we conducted multiple subgroup analyses. While these analyses provide exploratory insights, the large number of statistical tests increases the possibility of false-positive findings due to multiple comparisons. Although we additionally examined interaction terms, these findings should still be considered exploratory and require confirmation in future studies. Fifth, our analysis was limited to participants with retinal imaging data, which were only available in certain NHANES cycles and age groups, and some images were excluded due to poor quality. These exclusions reduced the sample size and may introduce selection bias. Sixth, the number of late AMD patients was less.

## 5. Conclusions

These findings provide epidemiological evidence that high nitrate and thiocyanate levels are associated with AMD risk. These exploratory findings are hypothesis-generating and highlight the need for further longitudinal and laboratory studies to clarify potential mechanisms and clinical implications.

## Supporting information

S1 Table Demographics of excluded and included participants. (PDF)
