# Supplementary material for: Association of perchlorate, nitrate, and thiocyanate with age-related macular degeneration in the United States
Source: PLoS One. 2025 Oct 29;20(10):e0334919. doi: 10.1371/journal.pone.0334919 (PMC12571269; doi:10.1371/journal.pone.0334919)
Supplement: S1 Table — (PDF) [file pone.0334919.s001.pdf]

S1 Table. Demographics of Excluded and Included Participants.

	All participants (n=20497)	Included (n=4727)	Excluded (n=15770)	P-value
Age, mean (SD), y	36.36 (22.17)	55.93 (11.43)	26.43 (19.54)	<0.001
Sex, No. (%)				0.135
Male	10176 (49.0)	2397 (48.0)	190 (49.4)	
Female	10321 (51.0)	2330 (52.0)	172 (50.6)	
Race, No. (%)				<0.001
Non-Hispanic White	8043 (67.3)	2604 (78.2)	256 (61.8)	
Non-Hispanic Black	4921 (12.3)	925 (8.9)	34 (14.0)	
Others	7533 (20.4)	1198 (12.9)	72 (24.1)	
